# Breaking the Dogma of Intravenous Treatment for Infective Endocarditis: A Systematic Review and Meta-Analysis

**DOI:** 10.3390/jcm13247518

**Published:** 2024-12-10

**Authors:** Beatrice Barda, Christian Schindler, Enos Bernasconi, Marco Bongiovanni

**Affiliations:** 1 Division of Infectious Diseases, Ente Ospedaliero Cantonale, 6900 Lugano, Switzerland; beatrice.barda@eoc.ch (B.B.); enos.bernasconi@eoc.ch (E.B.); 2 Swiss Tropical and Public Health Institute, 4051 Basel, Switzerland; christian.schindler@swisstph.ch; 3 Department of Biomedical Sciences, University of Southern Switzerland, 6900 Lugano, Switzerland

**Keywords:** infective endocarditis, oral antibiotic therapy, switch to oral treatment

## Abstract

**Introduction:** The treatment of infective endocarditis (IE) is based on long intravenous administration of antibiotics. This is still a hard-to-die dogma. Throughout the years, different researchers have attempted to demonstrate the safety and efficacy of an oral switch of the antibiotic regimen, with only scarce success. Nevertheless, in recent years, different reports have evaluated the efficacy of oral switch therapy in selected patients. Due to the lack of large trials, a meta-analysis could be useful to evaluate the potential benefits of early oral switch therapy not only in terms of microbiological cure but also in terms of relapse, mortality, and length of hospital stay (LOS). **Methods:** We conducted a Medline search, from which we were able to extrapolate 29 papers on IE treatment; the inclusion criteria were met by six papers only. Three trials were not randomized studies; therefore, we conducted the analysis both including and excluding the mentioned papers. **Results:** Overall, we conducted our analysis on 840 patients who received intravenous treatment and 677 who received oral treatment. Our results confirmed that oral switch therapy represents an option in selected patients, with a reduction in the relapse rate (OR: 0.54, 95% CI: 0.31–0.92). No statistically significant differences were observed for treatment failure (OR: 0.74, 95% CI: 0.48–1.14), length of hospitalization (OR: −0.42, 95% CI: −1.55–0.71), complication rate (OR: 0.38, 95% CI: 0.1–1.54), and mortality (OR: 0.52, 95% CI: 0.22–1.22). **Discussion:** Our results allow us to conclude that oral switch therapy is a feasible option in clinically stable patients with infective endocarditis. Moreover, oral switch therapy seems to perform significantly better than intravenous treatment in terms of relapse of infection. The data further support the implementation of oral switch therapy in infective endocarditis.

## 1. Introduction

Since the discovery of antibiotics, the treatment of infective endocarditis (IE) has been based on the prolonged intravenous (IV) administration of antibiotics, typically for a duration of 4 to 6 weeks. This approach arose from the understanding that IE pathogens colonize cardiac tissue, which represents a challenging site for both antibiotics and the immune system to penetrate effectively. This therapeutic approach developed at a time when penicillin was the most effective antibiotic available for IE treatment, and it was exclusively administered intravenously due to unreliable absorption when taken orally.

In the early 1930s, a study using oral sulfonamides for IE treatment yielded poor outcomes, with a cure rate of only 1%, which was in stark contrast to the 85% success rate achieved with parenteral penicillin G [1]. Nevertheless, the concept of oral therapy (PO) for IE persisted, and various trials have attempted to achieve effective results using a range of antibiotics. While antibiotics like macrolides, oral tetracyclines, and sulfonamides failed to reach the effective blood concentrations necessary for bactericidal action, some newer oral antibiotics appear capable of maintaining adequate concentrations in the bloodstream to combat IE pathogens. Although oral formulations of penicillin became available by the mid-1950s, they were not viewed as reliable alternatives for IE therapy due to concerns about inconsistent bioavailability and the historical failures of other oral antibiotics in achieving IE cures. Consequently, skepticism about the effectiveness of oral antibiotic therapy for IE has been prevalent since the 1950s.

There are, however, several reasons why oral antibiotics may still be viable options for treating IE. First, effective therapies are those that achieve and sustain adequate drug concentrations at the infection site, regardless of the administration route. Therefore, the critical factor is whether orally administered antibiotics can be absorbed at levels high enough to eradicate bacteria at the cardiac site of infection. Second, the low efficacy of older antibiotics, such as oral tetracyclines, macrolides, and sulfonamides, in IE is explainable by their inability to reach therapeutic levels in the blood and tissues [2,3,4,5]. By contrast, certain newly developed oral antimicrobial agents can achieve concentrations above the minimum bactericidal levels necessary to eliminate IE pathogens in the blood [6,7,8]. Supporting these pharmacokinetic findings, numerous randomized clinical trials (RCTs) have demonstrated that these agents, when administered at proper dosages, can successfully sterilize the blood in cases of Gram-positive cocci bacteremia.

Despite antibiotic availability, IE continues to be associated with elevated mortality rates, which are primarily attributed to heart failure resulting from valve damage [9]. The combination of surgical intervention with antibiotic treatment has significantly improved the prognosis of patients with IE; moreover, different studies have demonstrated the efficacy of surgery for patients with active IE experiencing complications, such as heart failure or uncontrolled infection [10,11,12]. Recently, another paper including individuals with IE and indication for cardiothoracic surgery showed that the presence of both heart failure and peripheral septic embolization worsen the prognosis of patients with IE, thus highlighting the importance of identifying specific features associated with poor prognosis in this heterogeneous setting of high-risk patients [13].

Currently, very few randomized controlled trials have directly compared outcomes in patients receiving exclusively IV treatment with those who switched to PO treatment after a brief IV course in IE cases. To gain further insight into this important topic, a meta-analysis including data from all available RCTs in the literature is essential. The results of our meta-analysis could improve the understanding of the potential benefits and risks associated with an early switch to PO treatment, not only concerning clinical and microbiological cure rates but also in terms of adverse events and hospital stay duration.

## 2. Methods

**Figure 1:**
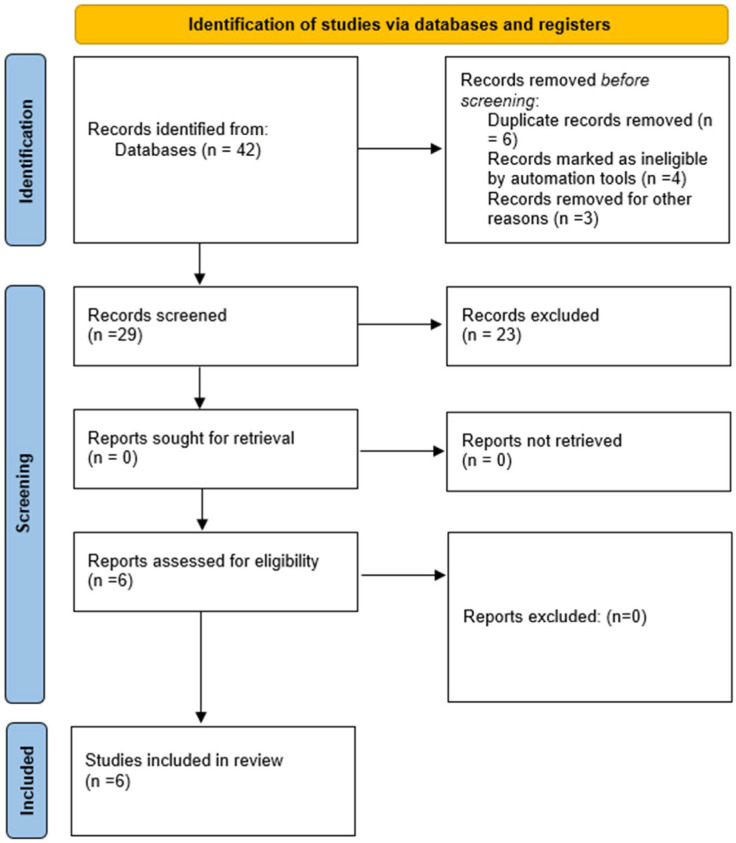
Prisma flow diagram for included studies.

We conducted an online search (based on PRISMA guidelines) using PubMed, Scopus, and Medline. We found 29 papers on infective endocarditis treatment that included the switch from a standard IV regimen to an early PO one. Fifteen papers were excluded from the meta-analysis because they were reviews, one was a meta-analysis, one was a comment to the POET study [14], one [15] is currently ongoing, one [16] analyzed secondary data of the POET study, and four [17,18,19,20] were retrospective studies (Figure 1).

Our meta-analysis included six studies [21,22,23,24,25,26], of which three [24,25,26] were not randomized trials. Therefore, the analysis was conducted both including and excluding these studies. The literature analysis was performed independently by BB and MB.

The outcomes were not standardized in the studies included in our meta-analysis. In the paper by Stamboulian et al. [21], the response of patients to therapy was clinically evaluated by daily evaluations of changes in signs and symptoms and for possible adverse reactions to the antibiotic that was administered. Relapse of infection was defined as the occurrence of signs and symptoms of infective endocarditis and cultures of blood positive for streptococci within 6 months of completion of antimicrobial therapy. In the paper by Heldman et al. [22], treatment failure was defined as the development, despite antibiotic therapy, of any of the following: left-sided endocarditis; heart block of any degree; congestive heart failure; respiratory failure requiring mechanical ventilation; hypotension; sustained or recurrent bacteremia after 10 days of treatment or during the inpatient antibiotic washout period or at outpatient follow-up assessment; metastatic infection, including secondary meningitis or osteomyelitis caused by the same microorganism; or death. Treatment success was defined as having blood cultures negative at 7 and 35 days after the end of the treatment. In the POET trial [23], the primary outcome was a composite of all-cause mortality, unplanned cardiac surgery, embolic events, or relapse of bacteremia with the primary pathogen from the time of randomization until 6 months after antibiotic treatment was completed. The primary outcome of the paper by Freling et al. [24] was a clinical success at 90 days, defined as being alive and without recurrence of bacteremia or treatment-emergent infectious complications, which were defined as infective conditions not present at the time of diagnosis that subsequently developed while on antimicrobial therapy. Such complications included the development of major arterial emboli, septic pulmonary emboli/infarcts, mycotic aneurysms, Janeway lesions, distant organ abscesses (e.g., liver/spleen/renal), visceral organ infarcts (e.g., splenic/renal), or distal organ hematogenous seeding (e.g., osteomyelitis). In the paper by Mzabi et al. [25], the status on the last evaluation was defined as alive with no relapse and/or reinfection, relapse, reinfection, or death. Relapse was defined by a new episode of endocarditis with the same microorganism and reinfection as a new episode of IE with a different microorganism. Finally, in the paper by Tissot-Dupont [26], the primary efficiency endpoint was mortality (global, at day 30 and at day 90); this paper also evaluated the length of stay in hospital, causes of death within 30 and 90 days, and the emergence of acute renal insufficiency.

To better evaluate such different outcomes and make the analyses as homogenous as possible, we performed different analyses grouping the studies according to the different outcomes considered. Therefore, the outcomes considered in our meta-analysis were treatment success [21,22,23,24], mortality at day 90 [21,24,25,26] and at 6 months after the end of treatment [23], relapse rate [21,23,25,26], treatment-related complications [22,23,24,25], and mean length of hospitalization [22,23,24,25].

## 3. Statistical Analysis

Meta-analyses were performed using Stata’s meta command, with random study effect variances being estimated using restricted maximum likelihood. These calculations rely on the absolute frequencies of positive and negative outcomes observed in the two arms across different studies. The results are presented as Forest plots containing detailed information, including the I-squared statistic. For binary outcomes, the summary estimate is reported as the odds ratio between the PO switch and IV-only treatments with a 95% confidence interval. To estimate a potential association between mean length of hospital stay (mLOS) and type of treatment, mLOS and its standard error were first estimated for each study and each study arm separately. In the second step, the difference in the natural logarithms of the mLOS between the PO and IV groups and its standard error was estimated for each study. Finally, these estimates were subjected to meta-analysis to obtain a summary estimate of the difference in the logarithmized mean ratio (i.e., of the natural logarithm of the mLOS ratio, referred to as LMR) between the PO and IV groups and its 95% confidence interval (see the Supplementary Materials for details).

## 4. Results

### Systematic Review of the Study Included in the Meta-Analysis

The first randomized trial addressed the possible role of oral antibiotics in the treatment of IE was performed by Stamboulian et al. in 1991 [21]. The authors randomized 30 patients with streptococcal left-sided endocarditis to either four weeks of IV treatment with ceftriaxone or two weeks of IV treatment (ceftriaxone 2 g/24 h), followed by two weeks of oral amoxicillin (1 g/6 h). All patients included were cured both clinically and microbiologically. The possibility of antibiotic administration on an outpatient basis was also an end-point, and the authors found that this possibility led to a significant reduction in hospitalization time with an overall reduction of 380 days [21]. However, the small number of subjects included represents a significant limitation of this study and can limit its applicability on a large scale.

A few years later, Heldman et al. [22] conducted a randomized, non-blinded, trial on intravenous drug users with a possible right-sided staphylococcal IE. Oral treatment included the association of ciprofloxacin (750 mg/12 h) and rifampin (300 mg/12 h), whilst IV treatment was oxacillin (2 g/4 h) or vancomycin (1 g/12 h) for penicillin-allergic patients, plus gentamicin 2 mg/kg/8 h for the first five days. If clinically contraindicated, vancomycin was substituted by cefazolin; the antibiotic dose was adjusted according to renal function. Eighty-five bacteremic subjects satisfied the criteria for at least possible right-sided staphylococcal endocarditis and were randomized to receive oral (n = 40) or intravenous treatment (n = 45). There were a total of four treatment failures (one in the oral and three in the intravenous group); however, the IV group experienced more adverse events compared to the PO group (62% vs. 3%, *p* < 0.0001), mainly due to oxacillin-related increase in liver enzymes [22].

The POET study is a large randomized controlled trial focused on treatment step-down in patients with uncomplicated, left-sided IE caused by *Staphylococcus aureus*, coagulase-negative staphylococci, streptococci, or *Enterococcus faecalis* [23]. All patients received at least 10 days of IV treatment and, if clinically stable (good clinical and microbiological response, negative blood cultures, and no signs of abscess at trans-esophageal echocardiography), were randomized to either continue the IV treatment (n = 201) or switch to PO treatment (n = 199). Intravenous treatment was decided according to the guidelines of the European Society of Cardiology; the oral regimens were based on pharmacokinetic calculations and expected minimal inhibitory concentrations for the isolated bacteria. Patients randomized to PO treatment were discharged to the outpatient clinic when feasible. The primary outcome was a composite of all-cause mortality, unplanned cardiac surgery, embolic events, or relapse of bacteremia with the primary pathogen from the time of randomization until 6 months after antibiotic treatment was completed. The primary composite outcome occurred in 24 patients (12.1%) in the IV-treated group and in 18 (9.0%) in the PO group, which met non-inferiority criteria. The switch to PO treatment was carried out on about day 17, meaning that these patients were eligible for partial or complete outpatient management. The results were consistent across different subgroups, including the type of valve affected (native or prosthetic valve) and the type of treatment (surgery during the disease course or conservative treatment). Furthermore, the primary outcome was comparable across the four bacterial types. Despite some limitations (patients included had endocarditis of the left-side heart, only some types of bacterial species were eligible, a limited population had *S. aureus* infection, and no patient had methicillin-resistant *S. aureus*), this trial represented a milestone in the therapeutic management of IE, putting the treatment of IE into a new spotlight and triggering the debate on the potential benefits of an early switch to PO therapy.

Freling et al. [24] conducted a retrospective study on 257 patients with IE diagnosed according to Duke’s criteria (two major criteria or one major and three minor criteria, or clinical suspicion documented by the physician). All of the subjects initiated an IV treatment and 46 of them transitioned from IV to PO therapy after a median time of 15.5 days. Patients in the IV arm were significantly older and had a higher proportion of aortic valve endocarditis, diabetes, dialysis dependence, and central venous catheters at the time of diagnosis. Conversely, the PO arm had significantly higher proportions of tricuspid valve endocarditis, subjects with a history of injection drug use, and infections by methicillin-resistant *Staphylococcus aureus* (MRSA). *Staphylococcus aureus* was the predominant pathogen in both groups, with 52.1% vs. 63.0% in the IV-only vs. PO arms, and also MRSA cases were predominant in the PO arm. Both IV and PO therapy were not standardized; therefore, several different regimens were used. The rates of clinical success (defined as being alive, without recurrent bacteremia, and without treatment-emergent infectious complications within 90 days) were similar between the groups; furthermore, there were also no significant differences in the individual components of the clinical failure composite definition (death, recurrence of bacteremia, and treatment-emergent complications) or 90-day readmission rates between the two groups. Finally, a similar proportion of patients in both groups failed to complete the planned duration of therapy (7.1% in the IV-only group and 6.5% in the PO group).

Mzabi et al. [25] carried out a retrospective cohort study over a period of 13 years (from 2000 to 2012) on patients with definite endocarditis (diagnosis made according to Duke’s criteria), of which 212 received IV therapy only, while 214 were switched to PO antibiotic after a median of 21 days from the diagnosis. Patients who switched to PO treatment had fewer comorbidities, met fewer criteria of severity at inclusion, and were less frequently infected by *S. aureus*. The switch was towards amoxicillin alone in 109 cases (50%) and towards a combination of clindamycin, fluoroquinolones, rifampicin, and/or amoxicillin in 46 cases (22%) according to microorganism susceptibility. In this paper, no difference was found in terms of increased risk of mortality in subjects switching to PO treatment. Furthermore, the risk of relapse or reinfection was comparable between the groups.

Tissot-Dupont et al. [26] conducted a retrospective cohort study on patients with *S. aureus* IE treated with IV antibiotics only or who stepped down to PO antibiotics after 7 days. From February 2012, patients with a diagnosis of IE by *S. aureus* (n = 171) received an antibiotic treatment including the combination of a high dose of trimethoprim–sulfamethoxazole (960 mg/4800 mg per day in six daily intravenous administrations, with the maximal dose adapted for weight and renal function) and clindamycin (1800 mg/daily in three intravenous administrations) for 7 days. After this initial IV lead, treatment was switched to oral trimethoprim–sulfamethoxazole 160 mg/800 mg alone (six tablets per day for a 5-week period, adapted for weight and renal function). If a complication occurred (positive blood cultures after 48 h or cardiac abscess), a combination of intravenous rifampicin (1800 mg/day) and gentamicin (180 mg/day) was added for 7 days. These patients were compared with a control group (patients admitted in the same clinic from December 2001 to January 2012, n = 170), who received a standardized antibiotic treatment: oxacillin 12 g/day intravenously for six weeks for methicillin-sensible *S. aureus* or vancomycin 30 mg/kg/day intravenously for six weeks for MRSA. This antibiotic therapy was combined with one daily injection of 3 mg/kg of gentamicin for five days. Based on the intention-to-treat analysis, the global mortality rate was lower in the PO group (19.3% vs. 30%; *p* = 0.024), as well as the in-hospital mortality (9.9% vs. 18.2%; *p* = 0.03) and the 30-day mortality (7.1% vs. 14.2%; *p* = 0.05). The mean duration of hospital stay alive was also significantly shorter in the PO group (30 vs. 39 days; *p* = 0.005).

## 5. Meta-Analysis

Overall, the data analyzed included 840 patients who received IV treatment and 677 patients who received PO treatment. We describe in the meta-analysis only the results obtained for comparable outcomes: treatment failure, relapse, complications, length of hospital stay, and death rate.

**Figure 2:**
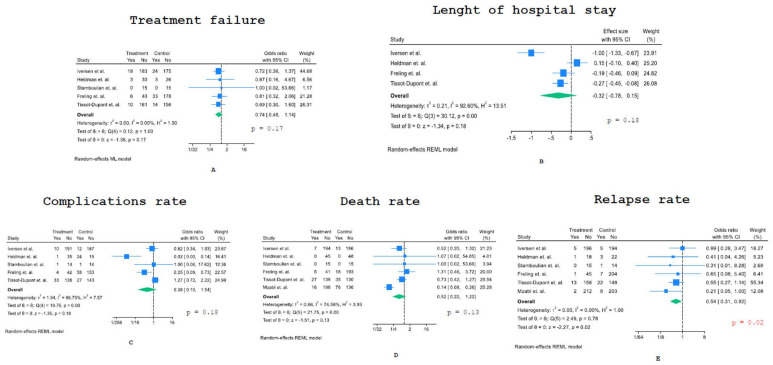
Forest plots of the meta-analysis: (**A**) treatment failure, (**B**) length of hospital stay, (**C**) complications, (**D**) death and (**E**) relapse rate [21,22,23,24,25,26].

As the Forest plots of Figure 2A show, the OR of treatment failure was 0.74 (95% CI: 0.48–1.14), not showing statistically significant differences between the PO and IV groups.

Figure 2B shows the Forest plot of the meta-analysis evaluating the length of hospital stay (LOS); we considered the logarithmized mean ratio (LMR) of LOS between the PO and IV groups as a measure of effect. On average, the mean LOS was shorter in the PO group than in the IV one, but this difference did not reach statistical significance (LMR–0.32; 95% CI: −0.78–0.15).

We then considered the rate of complications related to PO switch therapy versus IV treatment only (Figure 2C). A large and highly statistically significant difference was reported in Heldman et al.‘s paper [22], with only 1/36 patients experiencing an adverse event in the PO group compared to 27/39 patients in the IV group. Therefore, we calculated the OR of complications both including and excluding this study; the odds ratio was not statistically significant in both cases (OR 0.38, 95% CI: 0.1–1.54 and OR 0.65, 95% CI: 0.31–1.36, respectively).

The analysis of the death rate provided an OR of 0.52 (95% CI: 0.22–1.22), meaning that the early switch to PO treatment was associated with a lower average mortality rate, although this decrease was not statistically significant (Figure 2D). There was strong heterogeneity across studies, with a large and significant decrease in death rate in the PO switch arm of the study by Mzabi et al. [25].

All studies included showed a lower relapse rate under PO treatment compared to IV-only treatment (Figure 2E), providing an overall significant statistical difference (OR 0.54, 95% CI: 0.31–0.92, *p* = 0.02).

Finally, we performed statistical analysis only including the three randomized studies [21,22,23] and found that treatment failure (OR 0.74, 95% CI: 0.41–1.34), relapse rate (OR 0.74, 95% CI: 0.26–2.10), LOS (LMR0 −42, 95% CI: −1.55–0.71), complication rate (OR 0.26, 95% CI: 0.03–1.96), and death (OR 0.55, 95% CI: 0.23–1.35) were lower in patients who underwent PO switch therapy compared to the IV-only treatment. However, the confidence intervals of these summary odds ratios are extremely wide due to the small number of studies considered, the high uncertainty of the results in the studies of Heldman and Stamboulian [21,22], and/or the presence of significant heterogeneity (complications, LOS), thus limiting the robustness of these findings (Figure S1, Supplementary Materials).

## 6. Discussion

The existing literature provides limited evidence that an early switch to PO treatment is feasible for infective endocarditis. This meta-analysis represents the first comprehensive study to examine and analyze the current literature on IE treatment through a switch from IV to PO administration of antibiotics, particularly considering its safety, effectiveness, and applicability in diverse patient groups.

Several reviews have examined different antibiotic regimens for IE since the early 1970s [27,28,29]. However, there is a significant lack of robust evidence in this area. Randomized controlled trials are difficult to conduct due to the complex nature of IE, requiring careful patient selection to achieve optimal clinical outcomes and generate reliable data. In recent years, the POET trial marked a significant milestone by demonstrating that, in a specific subset of patients, an early switch from IV to PO treatment was non-inferior to extended IV therapy. This was particularly notable regarding six-month all-cause mortality, adverse event rates, and relapse rates of IE. However, it should be noted that patients included in the POET trial were in a stable clinical condition, with no complications related to IE, and had a controlled source of infection. Given the complexity of IE, many patients in real-world clinical practice do not fulfill these criteria, making it challenging to replicate these results broadly. Despite these limitations, the promising long-term outcomes from the POET study [30] have been incorporated into the European Society of Cardiology (ESC) guidelines [31]. The ESC guidelines now suggest that, in cases where the patient’s clinical condition is stable, outpatient parenteral antibiotic therapy (OPAT) or step-down oral antibiotic treatment could serve as a safe alternative to prolonged in-hospital IV therapy for selected patients.

The OPAT regimen involves the administration of the same antibiotic combinations used during the acute phase, where feasible, providing continuity of treatment outside the hospital setting. Clinical stability is essential to determine eligibility for PO treatment, with trans-esophageal echocardiography acting as a vital tool in assessing a patient’s readiness for this transition. This stability criterion ensures that only patients who have reached a level of infection control and have no underlying complications can safely switch to oral treatment.

Our meta-analysis suggests that an early switch to PO treatment for IE does not lead to increased rates of relapse, mortality, or treatment-related complications when compared to persistent IV therapy. Moreover, we observed a shorter average length of hospital stay in patients who underwent an early switch to PO treatment. This outcome not only points to the theoretical benefits of PO therapy in terms of reduced hospitalization costs and improved patient comfort but also raises the possibility of improving hospital resource utilization by freeing up beds for other critically ill patients.

These findings suggest that an early switch to PO therapy may be feasible in IE, provided patients meet specific criteria: the causative bacteria must be susceptible to the selected antibiotic; the patient must be in stable clinical condition; there should be no indication for cardiac surgery; blood cultures should be negative during follow-up assessments; the gastrointestinal tract must be intact to allow oral absorption; and psychological factors should support adherence to an oral regimen. In specific cases, such as with active intravenous drug users where discharge with a blood line is not an option, a fully oral regimen could be a practical alternative [22]. In an older study by Dworkin et al. [32], right-sided S. aureus IE was successfully treated in 14 intravenous drug users who received oral ciprofloxacin and rifampicin after a 7-day IV regimen of ciprofloxacin. All patients who completed the therapy (n = 10) were cured, and none experienced an infection relapse. Although additional evidence is needed, these data suggest that oral treatment could be a valuable alternative for patients unable to continue IV therapy for extended periods.

Another challenge lies in the significant variation in antibiotic regimens across studies, as well as changes in clinical guidelines over time. Consequently, it is difficult to directly compare treatments prescribed in the 1990s with those recommended in 2020. This variation emphasizes the need for clear and updated guidelines to support clinicians in selecting the most appropriate PO regimens for IE, tailored to individual patient needs.

To confirm these findings and better define the patient populations that would benefit from early PO switch therapy, larger randomized controlled trials with representative samples of IE patients are essential. These studies should aim to establish definitive clinical criteria and explore a wide array of patient conditions to determine the safest and most effective approach for transitioning from IV to PO therapy in the treatment of IE.

## 7. Limitations

Despite these encouraging findings, our meta-analysis has limitations that hinder the ability to make definitive conclusions. First, few randomized clinical trials have directly compared the effectiveness of IV-only treatment versus PO treatment in terms of mortality, clinical and microbiological cure, infection relapse, LOS, and treatment-related adverse events. Therefore, most of the data that we analyzed stemmed from observational studies or older studies with limited methodological rigor. Additionally, outcome definitions varied among studies; for instance, mortality was assessed at different time intervals, and definitions of “cure” or “relapse” lacked standardization. This lack of uniformity extends to adverse events as well, with inconsistencies in their classification and reporting. Standardized outcome definitions and consistent study designs in future research would greatly enhance the reliability of findings and improve comparability between studies.

Another significant limitation is that patients selected for a step-down from IV to PO treatment were predominantly those in stable clinical conditions. Typically, the infection source was under control, and no major complications were present at the time of the switch, which likely skewed outcomes for the PO group. This selection bias raises questions about the applicability of these results in a wider clinical setting, where patients may have varying degrees of clinical stability and associated complications. In such cases, the transition from IV to PO therapy may not yield equivalent results in terms of mortality and efficacy, thus limiting the generalizability of our findings to broader clinical practice.

A further limitation, noted in previous reviews as well as in our meta-analysis, is the variability in the duration of the IV lead-in phase across studies. The discrepancies in IV lead duration pose a challenge in reaching a clear conclusion regarding the safety and efficacy of an early switch to PO treatment. For example, the IV treatment duration in the Mzabi study [25] was 21 days, while the POET trial [23] employed an IV duration of only 7 to 10 days before switching to oral therapy. In Freling et al.‘s study, only patients who had received at least 14 days of IV antibiotics were eligible for PO switch therapy [24], whereas in the Heldman study, patients were transitioned directly to PO therapy without an initial IV period [22]. Freling et al. also examined clinical success after varying IV lead durations (<7 days, 8–14 days, 15–21 days, and >21 days), finding no significant differences in clinical success (measured at both 90 days and final follow-up assessment) or in the 90-day readmission rate [24]. A retrospective analysis by Demonchy [33] further supported PO therapy, noting lower mortality in patients who switched to PO treatment after an 18 + 9-day IV period (0% vs. 21%, *p* = 0.052).

Therefore, at the moment, all of these limitations make PO switch therapy a challenging option in the management of patients with IE.

## 8. Impact on Antimicrobial Stewardship Programs

One of the primary objectives of antimicrobial stewardship programs is to promote the switch of antibiotics from intravenous to oral treatment. In modern medical practice, there is an increasing emphasis on optimizing antibiotic use to minimize the risk of resistance and adverse side effects while maximizing patient outcomes. The transition to PO therapy is seen as a crucial component of this optimization strategy, especially given the growing body of evidence supporting its safety and effectiveness in treating various infections.

However, some clinicians continue to adhere to the traditional approach of prolonged IV treatment, viewing it as safer due to its historical use and the perception of reliability associated with this method. This conservative mindset is rooted in the belief that IV antibiotics offer more immediate therapeutic effects, which can be crucial for managing severe infections. The reluctance to switch early to PO therapy, despite the increasing evidence in favor of this practice, reflects a cautious approach to treatment. Frequently, PO switch therapy is regarded as a mere simplification of therapy rather than an opportunity to select the most appropriate narrow-spectrum antibiotic. This perspective fails to recognize the clinical advantages that can arise from the judicious use of oral agents.

A paradigm shift is necessary, particularly at the hospital level, to encourage the adoption of early oral therapy where feasible. Infectious disease specialists play a vital role in this transition by advocating for the responsible use of antibiotics and discouraging reliance on broad-spectrum agents administered intravenously for extended periods. By doing so, they can help mitigate the risks of antibiotic resistance and associated complications. New therapeutic approaches are emerging that promote the early use of oral therapy, even for infections historically treated solely with IV antibiotics, such as endocarditis or bone and joint infections. The goal is to reduce the length of hospital stays for patients with difficult-to-treat infections while ensuring they receive adequate and effective antibiotic therapy.

In recent years, innovative scoring systems have been proposed to evaluate the spectrum of antibiotic treatments [34,35,36,37]. These spectrum scores assign numeric values to individual antibiotic agents that correspond to their unique spectra of activity. Such systems aim to objectively quantify the activity of antibiotics, providing a standardized classification that can aid clinicians in making informed decisions regarding treatment options. Moreover, these numeric values can be combined with other variables to develop a comprehensive score that assesses the spectrum of a specific antibiotic treatment.

One notable example is the score proposed by Madaras-Kelly et al. (MKSS), which was designed to detect antibiotic de-escalation events [34]. This score includes PO switch therapy as a variable that can potentially lead to improvements in various clinical measures, such as the length of stay (LOS), the rate of treatment-related complications, and more appropriate antibiotic use. Despite the broad potential applications of these scores, the lack of standardization, the different variables considered, and the variations in the relative ranking of antibiotics within each score make large-scale interpretation and application challenging. Nevertheless, an ideal score to evaluate the antibiotic spectrum should incorporate oral switch therapy, the duration of treatment, and the risk factors for multidrug-resistant bacteria (such as recent hospitalization or the prevalence of resistant bacteria in a specific geographical area), in conjunction with the score for each individual antibiotic. Implementing these scores in the current clinical practice for a broad spectrum of infections (including those requiring prolonged treatment such as endocarditis or osteomyelitis) could lead to better evaluation of the consumption of antibiotics at hospital levels and thus optimize their use and support the clinical implementation of the early switch to PO therapy.

In conclusion, the transition from IV to oral antibiotic therapy should be more widely adopted within antimicrobial stewardship programs. Emphasizing the clinical benefits of early PO therapy can help optimize patient outcomes, reduce hospital stays, and minimize the risks associated with prolonged IV treatment. The integration of innovative scoring systems could facilitate this transition by providing a structured approach to assessing the appropriateness of antibiotic regimens. Ultimately, fostering a culture that prioritizes effective antibiotic use, while also considering patient safety and treatment efficacy, is essential for advancing the field of infectious disease management.

## 9. Conclusions

Our meta-analysis aimed to summarize the existing evidence regarding the potential benefits and risks associated with switching from intravenous to oral antibiotic therapy in patients diagnosed with infective endocarditis (Table 1). This condition, characterized by inflammation of the heart’s inner lining due to an infection, poses significant treatment challenges and often necessitates prolonged antibiotic therapy. Historically, the standard treatment was based on extended IV administration of antibiotics, which was primarily due to concerns about efficacy and safety. However, our findings indicate that an early switch to PO therapy is feasible in clinically stable patients, regardless of whether they present with right-sided or left-sided infective endocarditis.

Notably, our analysis also encompassed patients with various etiologies of endocarditis, including infections caused by Staphylococcus aureus—both methicillin-susceptible (MSSA) and methicillin-resistant (MRSA)—as well as streptococci. This broad applicability suggests that the oral switch strategy can be effectively implemented across a range of clinical scenarios, expanding treatment options for a diverse patient population.

The implications of this approach are particularly significant for patients who may face difficulties with IV access, such as intravenous drug users. For these individuals, transitioning to oral therapy not only addresses the challenges associated with maintaining a blood line but can also enhance adherence to prescribed treatment regimens. Improved adherence is crucial, as it directly correlates with better patient outcomes and overall prognosis.

Furthermore, our analysis revealed that the risk of developing adverse events during the transition to PO treatment is not negatively influenced by this switch, suggesting that PO treatments are overall safe alternatives. Interestingly, although the differences were not statistically significant, we observed trends indicating that PO switch therapy could potentially reduce the length of hospitalization and minimize side effects commonly associated with IV administration. This finding is particularly relevant given the healthcare system’s increasing focus on reducing hospital stays to enhance patient comfort and optimize resource utilization.

Moreover, we found that the risk of relapse in patients who switched to PO treatment was lower compared to those who continued with IV therapy. This further underscores the adequacy and effectiveness of oral antibiotics, providing additional support for the clinical implementation of early PO switch therapy in suitable patients. The ability to achieve comparable efficacy while potentially improving patient quality of life presents a compelling case for re-evaluating traditional treatment paradigms for infective endocarditis.

In conclusion, while there are few randomized trials that directly compare the efficacy of PO and IV treatments for infective endocarditis, the data currently available are consistent with the comparable effectiveness of the two approaches in selected individuals, particularly in settings where a well-established network of healthcare professionals exists. These findings were also more clearly in favor of an early oral switch when also including the observational studies. This is not surprising given a likely therapeutic bias in these studies that usually included individuals with adequate clinical and microbiological responses. The shift towards considering an early switch to PO therapy reflects a broader trend in antimicrobial stewardship, emphasizing the importance of optimizing treatment regimens to enhance patient outcomes while minimizing unnecessary risks associated with prolonged intravenous therapy.

## Figures and Tables

**Table 1:** Summary of the findings of our meta-analysis.

Studies evaluating oral switch therapy in patients with infective endocarditis are lacking, especially randomized controlled trials.
Lack of standardization of the outcomes evaluated in the different studies make it difficult to obtain solid data.
Our meta-analysis, though with the above-described limitations, is the first to try to offer a contribution to addressing the role of oral switch therapy in the treatment of infective endocarditis.
Our data showed that the relapse rate was lower in subjects switching to PO treatments.
No differences were observed in terms of treatment failure, length of hospital stay, complication rate, and death rate.
More data from randomized controlled trials with standardized outcomes are mandatory to finally assess the role of oral antibiotics in the treatment of infective endocarditis.

## Supplementary Materials

The following supporting information can be downloaded at: https://www.mdpi.com/article/10.3390/jcm13247518/s1, Figure S1: Forest plots of the meta-analysis inclyding only randomized studies: (**A**)treatment failure, (**B**) length of hospital stay, (**C**) complications, (**D**) death and (**E**) relapse rate.
