# Supplementary material for: Breaking the Dogma of Intravenous Treatment for Infective Endocarditis: A Systematic Review and Meta-Analysis
Source: J Clin Med. 2024 Dec 10;13(24):7518. doi: 10.3390/jcm13247518 (PMC11677701; doi:10.3390/jcm13247518)
Supplement: Supplementary file 1 [file jcm-13-07518-s001.zip › jcm-3318120-supplementary.pdf]

Supplement: Analysis of length of hospital stay

October, 2024

1 Estimation of mean and standard deviation from quartiles

Some of the papers analyzed provided group means and standard deviations of length of hospital stay (LOS), others the group-specific quartiles $LOS_{0.25}$, $LOS_{0.5}$ and $LOS_{0.75}$. Let $m(LOS)$ denote a group mean of LOS and $SD(LOS)$ its standard deviation. If the quartiles of LOS were given, we estimated $m(LOS)$ and $SD(LOS)$ under the assumption that LOS had a log-normal distribution. This provided

$$m(ln(LOS)) = ln(LOS_{0.5}) \quad (1)$$

$$SD(ln(LOS)) = \frac{ln(LOS_{0.75}) - ln(LOS_{0.25})}{2 \times 0.6745} \quad (2)$$

Notice that 0.6745 is the upper quartile of the standard normal distribution.
From there, one obtains the following estimates for $m(LOS)$ and $SD(LOS)$

$$m(LOS) = LOS_{0.5} \times exp(SD(ln(LOS))^2/2) \quad (3)$$

$$SD(LOS) = m(LOS) \times \sqrt{exp(SD(ln(LOS))^2) - 1} \quad (4)$$

The assumption of a log-normal distribution of LOS must be seen as a considerable limitation.

2 Approximate standard errors of relative differences in means

Let μ_1 and μ_2 and σ_1 and σ_2 denote the means and standard deviations of a continuous random variable X in two different populations, and assume that m_1 and m_2 are the means of X in two indpendent random samples of sizes n_1 and n_2 from the two populations. Moreover, let s_1 and s_2 denote the standard deviations of X in these samples.
We are interested in the statistic

$$T = ln(\frac{m_2}{m_1}) \quad (5)$$

Let $dm_1 = m_1 - \mu_1$ and $dm_2 = m_2 - \mu_2$ We then have

$$T = ln(\mu_2 + dm_2) - ln(\mu_1 + dm_1) \quad (6)$$

$$= ln(\mu_2) - ln(\mu_1) + ln(1 + \frac{dm_2}{\mu_2}) - ln(1 + \frac{dm_1}{\mu_1}) \quad (7)$$

$$\approx ln(\mu_2) - ln(\mu_1) + \frac{dm_2}{\mu_2} - \frac{dm_1}{\mu_1} \quad (8)$$

From this it follows that

$$SE(T) \approx \sqrt{\frac{\sigma_2^2}{n_2\mu_2^2} + \frac{\sigma_1^2}{n_1\mu_1^2}} \quad (9)$$

$$\approx \sqrt{\frac{s_2^2}{n_2m_2^2} + \frac{s_1^2}{n_1m_1^2}} \quad (10)$$

Of course, these approximations are valid only if the sample sizes are large enough. Despite all

limitations, we used formulas (1) to (4) and (10) to obtain estimates of T and $SE(T)$ for the studies of Freling and Iversen.

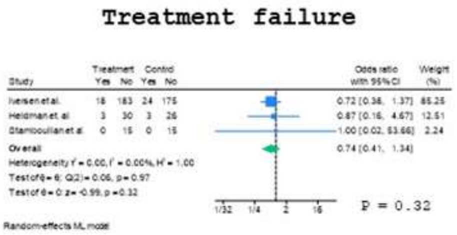

Figure 3A

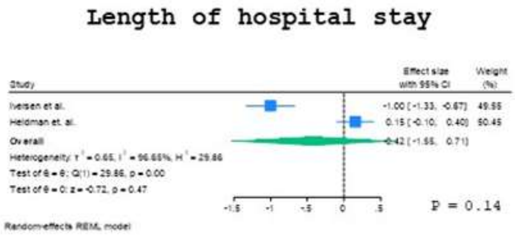

Figure 3B

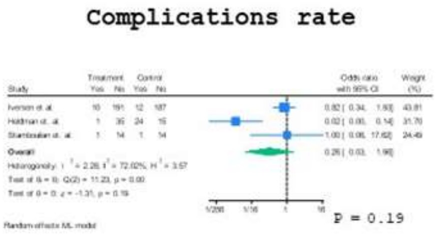

Figure 3C

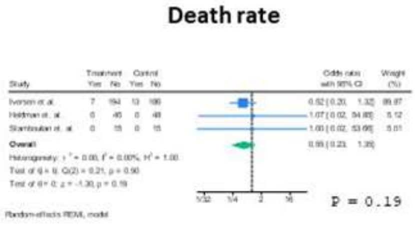

Figure 3D

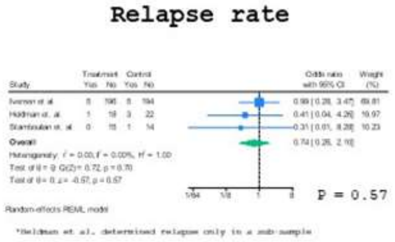

Figure 3E

Figure 3. Forrest plots of the meta-analysis including only randomized studies: A) treatment failure, B) length of hospital stay, C) complications, D) death and E) relapse rate.
